# Principles of Special and Comparative Physiology, Intended as an Introduction to the Study of Human Physiology, and as a Guide to the Philosophical Pursuits of Natural History

**Published:** 1839-04-13

**Authors:** 


### BIBLIOGRAPHICAL NOTICE.

*Principles of Special and Comparative Physiology, intended as an introduction to the Study of Human Physiology, and as a Guide to the Philosophical Pursuits of Natural History.* By Wm. B. Carpenter, Member of the Royal College of Surgeons, &c. &c. London: 1839. 8vo. pp. 470. With numerous Engravings on Copper.

Physiology has always been deeply imbued with the spirit of the dominant philosophy of the age—the scientia scientiarum, which has for its object the investigation of the principles of all human knowledge. Since Greece boasted a philosophy this has been the case. The theories of the ancient physiologists were grounded on and modified by the favourite metaphysics of the day. On the revival of letters, the same union was again effected, and continues until the present time. As it has been speculative or practical, so has been medicine. As it was sectarian, or eclectic, or catholic, so was medicine. The Italians, the Ionians, the Academicians, the Peripatetics, profoundly tinctured the medical dogmas of their age. Since the sixteenth century the doctrines of Descartes and Leibnitz, of Locke and Bacon, of natural or acquired ideas, as well as the gloomy and gorgeous mysticism of Kant, have either led to the grossest materialism and necessity, or to the idea of some subtle and incomprehensible vitalizing essence. Within a few years philosophy has assumed some determinate shape and purpose, and we accordingly find medicine partaking of the same spirit. Unfortunately, however, the vast mass of facts which ages should have collected in the great treasury of knowledge, is found to be so alloyed and debased, as to compel its cultivators to re-commence with the elements. That this portion of the labour will be properly conducted we may reasonably anticipate, and a slow but steady progress to truth must necessarily result.

The object of the ancient philosphy seems to have been the resolution of enigmas; a mental achievement which though it doubtless invigorated the intellects of the disputants, neither increased the comforts of the mass, or in any wise alleviated their miseries. Many of the most powerful and brilliant minds that any age could boast, were thus systematically perverted, and industry, ingenuity and zeal were absolutely wasted on the vaguest and most fruitless subtilties. There was continual and extraordinary exertion, but no corresponding progress. It transmitted to posterity no heritage of truth. To use the language of a profound and eloquent writer on that period, "every trace of an intellectual culture was there but the harvest. There had been plenty of harrowing, reaping, and threshing. But the garners contained only smut and stubble." An insatiable thirst for novelty as well as victory characterized their sages, and ingenuity, distortion, and garrulity were their chief weapons. There was nothing practical, nothing useful, proposed, or attained. "It was an intricate wood of briers and thistles, from which those who lost themselves in it brought back many scratches and no food."

How immensely different are the intentions and designs of modern philosophy—the extension of rational enjoyment; the mitigation of suffering. "A point which was invisible will be its goal to-day, and its starting place to-morrow."

Our chief care should now be to avoid all tendency towards a retrograde movement, which would prostrate the foundations which have been just reared, and which promise to be adequate to the support of the mighty edifice to be erected. We feel assured that no more effective barrier can be interposed than an early and deep devotion to the physical sciences among the builders of the temple. The elements of these are easily attained in early life, and by their mental training must exercise the happiest influence on the intellectual constitution of man. The collateral benefits of such a course of study is its rigorous mental discipline, rather than any appreciable amount of positive knowledge. It habituates the mind to habits of strict reasoning, and to the contemplation and comprehension of the most important and complex mental processes, familiarizing it with that peculiar conviction produced alone by demonstration, and excluding all considerations not entering therein. A habit of scrupulous and vigilant reasoning is thus engendered, becoming in time of incalculable advantage. Perhaps a moderate acquaintance with the principles of abstract science, is the most suitable introduction to the study of physics, as from their definite objects and distinct ideas we invariably learn and appreciate that precision of language as an instrument of reason, which materially lessens the chance of future error and confusion. It impresses one, moreover, with the distinction between strict and vague reasoning, giving one accurate and positive ideas concerning demonstrative evidence.

The legitimate object of scientific investigation, are the principles or laws regulating the phenomena we notice. The effects, or insulated facts, are subjects of daily observation to the most uninitiated. The province of the philosopher is to ascertain how they are determined and regulated. Art has been defined to be, the application of knowledge to a practical end. It is empiricism, when it is simply accumulated experience. It becomes a science, when submitted to the process of reason, and subjected to certain general principles.

Much has been recently said relative to the introduction of the self-styled Baconian philosophy into medicine; and a school has been erected, having for its avowed object the dissemination of principles, which, in the sanguine expectation of its members, are to lead medicine swiftly and surely to the long-sought land of promise. Much as we respect the intentions of this sect, and appreciate their labours, we feel ourselves called on to pronounce their claims as the only true *observers*, as unfounded, and their gratulatory cries of triumph as entirely premature. We do not regard M. Louis as the Moses of medicine, much less its Joshua, any more than the Hôtel Dieu as Mount Pisgah. We feel confident in the assertion that his followers will never reach the verge, much less penetrate the heart of Canaan. That work is, if we mistake not, reserved for another age. The materials may be collecting; but the present generation is not, we fear, to have the honour of giving the architect. Many wildernesses are yet to be traversed, many rapid and bitter streams to be crossed. We are at best in a state less of full development, than of happy progress. Let some definite object always be kept in our "mind's eye," which will stimulate us to the careful and accurate performances of the mental processes, and success will eventually crown our labours. The true philosophical spirit has been characterized as one of much hope and little faith.

Greek philosophy recognised the necessity of observations, and asserted their paramount value. But these steps alone did not lead to science. Aristotle expressly asserts, "the way must be the same with respect to philosophy, as to any art or science whatever. We must collect the facts, and the things to which the facts happen in each subject, and provide as large a supply of these as possible." (*Annal. Prior.* i. 30.) Mr. Whewell, in his admirable and philosophical history of the Inductive Sciences, has cited innumerable passages from the writings of the Stagyrite, in the tone and spirit of modern philosophy. Every mental process of the humblest intellect is always an induction, and conducts to truth or error according to the mode in which it has been performed—with accuracy or with carelessness. By induction, nature is said to be interpreted, and not anticipated. But, is not the anticipation, after all, but a slovenly interpretation, in which some vital element is overlooked? We do not believe that rules, no matter how strict, or systems, no matter how elaborate, will ever be a successful substitute for natural sagacity, judgment, and patience. The merits of the *observers* of our day we do not pronounce overrated, but only misunderstood.

In studying the history of science, we mark the development of human intellect from its simplest expression to its most intricate processes. These mental cycles seem to us to be best indicated and classified by M. Compte in his recent invaluable publication.* In its infancy, thirsting after a knowledge of final causes, the mind refers all the anomalies of creation to supernatural agents, who directly or remotely exert their influence. This is the age of superstition and theological tyranny. Next the metaphysical philosophy gives rise to the entities, or personified abstractions, inherent forces generated in the material universe, producing and regulating its phenomena. This is the state of transition, or chrysalis. Lastly arises the positive philosophy, to which science tends as its ultimate haven and abiding place. It abjures all profitless speculation about the origin or purpose of things, and, contenting itself simply by combining *reason* and *observation*, by inquiring what are the relations of succession and similitudes, endeavours to refer all things to various acts of some grand and general principle. Nowhere, perhaps, in the whole range of philosophical literature, is so admirable a practical illustration of the spirit of this doctrine given, as by Fourier in his *Theory of Heat*, where, without noticing either the intimate nature of heat, or the calorific sects, he enunciates precisely and fully the most important thermological laws.

An attentive perusal of Mr. Carpenter's work has afforded us the liveliest gratification, and induced us to hazard the present remarks. It is conceived and executed in a truly happy and philosophical spirit. It fills a sensible gap in English physiological literature, long deeply felt, and which the translation of Tiedemann's publication did not meet. We would earnestly recommend it to all students of physiology, and to all who intend to become students. We are convinced that a general survey of the analogies and intimate connexion between all animated nature forms the best introduction to the great object of his future studies—the last link in the organic chain.

Mr. Carpenter throughout this work exhibits an extensive and accurate acquaintance with human and comparative physiology and anatomy, as well as botany and zoology, and when we consider that the author is a young man, and a recent graduate, the highest anticipations of future excellence and ability may be rationally indulged. We have room only for a short extract from the preface, and to add that the literary character of the work is highly creditable.

"It is now generally acknowledged that physiology can only be properly studied by a constant reference to the comparative structure and functions of many different classes of animals; and in most of the recent works on this science, an outline of the development and actions of each system in the inferior tribes is prefixed to the details relating to its condition in man. This outline is filled up in the present volume, not only by amplifying the portion of it which relates to the animal kingdom, but also by the introduction of a similar view of the comparative structure and functions of vegetables, which is here shown to be governed by the same laws. It is this which constitutes the peculiar feature of the work; as the author believes it to be the first attempt, in this country at least, to form any thing like a systematic comparative physiology of vegetables. The translation of the elaborate comparative physiology of Tiedemann would, indeed, have occupied this ground; but it is still incomplete, and is likely to remain so; and the mass of details which it embraces, unconnected by comprehensive principles, renders it most tedious and embarrassing to the student. From that most valuable storehouse of *facts*, the present volume differs essentially, therefore, in plan; this being devoted to the explanation and illustration of general *laws*."

### CLINICAL LECTURE.

### PHILADELPHIA HOSPITAL.

ON PNEUMONIA—(CONTINUED.)

As an illustration of pneumonia, complicated with disease of the brain, I cannot do better than read to you the case of a young negro, of a slender frame, whom you have several times seen. It is an instance of arachnitis supervening towards the termination of the pneumonia, and therefore strictly secondary. You must have

* Cours de Philosophie Positive. Par M. Auguste Compte. 3 tom. Paris: 1830-5-8.

